# Doxycycline and Zinc Loaded Silica-Nanofibrous Polymers as Biomaterials for Bone Regeneration

**DOI:** 10.3390/polym12051201

**Published:** 2020-05-25

**Authors:** Manuel Toledano, Manuel Toledano-Osorio, Raquel Osorio, Álvaro Carrasco-Carmona, José-Luis Gutiérrez-Pérez, Aida Gutiérrez-Corrales, María-Angeles Serrera-Figallo, Christopher D. Lynch, Daniel Torres-Lagares

**Affiliations:** 1 Dental Materials Section, Faculty of Dentistry, University of Granada, Colegio Máximo de Cartuja s/n, 18071 Granada, Spain; toledano@ugr.es (M.T.); mtoledano@correo.ugr.es (M.T.-O.); alcarcar94@correo.ugr.es (Á.C.-C.); 2 Oral Surgery Section, Faculty of Dentistry, University of Sevilla, Avicena s/n, 41009 Sevilla, Spain; jlgp@us.es (J.-L.G.-P.); agcorrales@us.es (A.G.-C.); maserrera@us.es (M.-A.S.-F.); danieltl@us.es (D.T.-L.); 3 University Dental School & Hospital/University College Cork, Wilton, T12 E8YV Cork, Ireland; chris.lynch@ucc.ie

**Keywords:** bone regeneration, vascularization, macrophage, zinc, doxycycline, non-resorbable polymer, cells, silica

## Abstract

The main target of bone tissue engineering is to design biomaterials that support bone regeneration and vascularization. Nanostructured membranes of (MMA)_1_-co-(HEMA)_1_/(MA)_3_-co-(HEA)_2_ loaded with 5% wt of SiO_2_-nanoparticles (HOOC-Si-Membrane) were doped with zinc (Zn-HOOC-Si-Membrane) or doxycycline (Dox-HOOC-Si-Membrane). Critical bone defects were effectuated on six New Zealand-bred rabbit skulls and covered with the membranes. After six weeks, the bone architecture was evaluated with micro computed tomography. Three histological analyses were utilized to analyse bone regeneration, including von Kossa silver nitrate, toluidine blue and fluorescence. All membrane-treated defects exhibited higher number of osteocytes and bone perimeter than the control group without the membrane. Zn-HOOC-Si-Membranes induced higher new bone and osteoid area than those treated with HOOC-Si-Membranes, and control group, respectively. Zn-HOOC-Si-Membranes and Dox-HOOC-Si-Membranes attained the lowest ratio M1 macrophages/M2 macrophages. Dox-HOOC-Si-Membranes caused the lowest number of osteoclasts, and bone density. At the trabecular new bone, Zn-HOOC-Si-Membranes produced the highest angiogenesis, bone thickness, connectivity, junctions and branches. Zn-HOOC-Si-Membranes enhanced biological activity, attained a balanced remodeling, and achieved the greatest regenerative efficiency after osteogenesis and angiogenesis assessments. The bone-integrated Zn-HOOC-Si-Membranes can be considered as bioactive modulators provoking a M2 macrophages (pro-healing cells) increase, being a potential biomaterial for promoting bone repair.

## 1. Introduction

After tooth extraction, it is unavoidable to obtain a substantial alveolar bone loss. Therefore, bone regeneration procedures are required, in some instances, in dental and maxillofacial clinics to gain bone volume [1]. In bone tissue engineering, the design of materials that support formation and remodeling of bone tissue, as well as stimulating bone tissue to regenerate is a main objective [2]. Bone remodeling is the result of a series of biological events, regulated by the different bone cell types, mainly osteocytes, osteoblasts and osteoclasts [3]. In guided bone regeneration these cellular components are considered key factors [3]. During bone development, cell behavior and differentiation are coordinated by cell-cell communications. Therefore, in order to synchronize both angiogenic and osteogenic conditions, the cells type careful adjustment is an important parameter [4]. Newly formed capillaries do also play a vital role in the successful engineering of tissue constructs. 

The elementary principle of bone growth strategy, in guided bone regeneration, comprises the placement of mechanical barriers (membranes), in order to obtain the protection of blood clots and the isolation from surrounding connective tissue, thus, offering the access to a secluded space to the anterior bone-forming cells for bone regeneration and vascularization [5]. In order to warrant the bone remodeling and maturation, non-resorbable membranes are preferred to protect the newly-formed bone from physiological stress, and to exert spatio-temporal control over the wound-healing process [6]. There is a distinct lack of non-resorbable biomaterials with appropriate properties as an alternative to polytetrafluoroethylene (PTFE) membranes. They have low adhesiveness for plasma proteins and cells, a lack of osseointegration and inadequate topography resembling bone tissue [7].

In a previous study, we reported new polymeric and non-resorbable membranes, obtained by electrospinning, and loaded with calcium or zinc ions that have proven their efficacy for guided bone regeneration in rabbits calvarial defects [6]. To enhance hydrophilicity, cell-membrane interactions, mechanical properties, osteogenic and to confer antibacterial properties, a novel composite membrane based on the electrospun of a mixture of (MMA)_1_-co-(HEMA)_1_ and (MA)_3_-co-(HEA)_2_ doped with silicon dioxide nanoparticles (SiO_2_-NPs) are proposed. The composite membranes were loaded with silica nanoparticles to promote their integration with human tissues by inducing scaffold bioactivity, osteoconductivity [8] and facilitating surface formation of calcium phosphate deposits. The potential to drive the osteogenic differentiation of the progenitor cells is the most fascinating feature of the bioactive materials [2]. The presented membranes werefunctionalized with zinc or doxycycline trying to potentiate osteogenic and vascular responses [9], and to increase the mineralized and osteoid new bone formation [6]. A multi-parameter characterization was performed on these membranes showing a surface topography (fiber to fiber distances, fiber diameter and roughness) and surface mechanical properties similar to those of trabecular bone [10]. Doxycycline and zinc doping efficacy were demonstrated; static in vitro bioactivity and osteoblasts cells adhesion/proliferation were also stated [10]. Then, the experimental biomimetic membranes may be considered as a novel potential construct intended for enhancing bone regeneration processes.

Any biomaterial success or potential failure is influenced by the macrophages. The main macrophage phenotypic profile recognition can be a useful tool by to predict and possibly promote the tissue regenerative outcome [11]. Activated macrophages can be classified as pro-inflammatory (M1) or as anti-inflammatory or pro-healing (M2) cells [12]. The macrophages phenotypic profile as M1 or M2, i.e., modulation of macrophages polarization [13], following the exposition to a biomaterial can determine angiogenesis and remodeling of injured tissues [11]. Biomaterials-mediated bone formation success depends on a M1 to M2 phenotype efficient and timely switch during bone healing. The extension of a M1 phase can cause fibrous encapsulation and bone regeneration failure [13].

The objective of this research was to evaluate the regenerative potential of novel nanostructured membranes silica loaded and doped with zinc or doxycycline in a critical sized calvarial bone defect rabbit model. The null hypotheses to be proved were that: (1) The novel membranes doped with the bioactive agents do not facilitate bone regeneration; (2) the novel membranes doped with the bioactive agents do not modulate macrophages phenotype ratio (M1/M2) and do not influence both angiogenesis and osteogenesis.

## 2. Materials and Methods

### 2.1. Preparation of Membranes

Nanostructured membranes of hydrophilic nonwoven nanofibers, based on acrylate and methacrylate copolymers were manufactured by NanomyP^®^ (Granada, Spain). A novel polymer blend was used: (i) The first copolymer of (MA)_3_-co-(HEA)_2_ with statistical topology in a 50% by weight; and (ii) a second copolymer of (MMA)_1_-co-(HEMA)_1_ in a 50% by weight [14]. The first copolymer has a molecular weight of 1.9 × 10^6^ Da and the second copolymer has a molecular weight of 230,000 Da. For the synthesis of the first and second copolymers a metal-catalysed living radical polymerization and a reverse atom transfer radical polymerization were respectively used. The membranes were fabricated trough a electrospinning process of the final polymer blend [(MMA)_1_-co-(HEMA)_1_/(MA)_3_-co-(HEA)_2_]. The membrane further comprises 5% wt of SiO_2_ nanoparticles (NPs-SiO_2_), suspending them in the electrospinning solution and, then, the electrospinning process was carried out. SiO_2_ nanoparticles resulted homogenously dispersed in the membrane, trapped homogeneously in the whole fiber volume forming a solid solution (composite). To activate the surface of the membranes whit carboxyl groups (HOOC-Si-membrane) they were immersed for 2 h into a buffer solution of sodium carbonate (333 mM; pH = 12.5). Due to ester bonds partial hydrolysis, this process disposed carboxyl groups on their surfaces [15]; subsequently, membranes were gently washed in distilled water and dried in a vacuum oven [16]. Carboxyl groups ability to complex divalent cations was used to functionalize the membranes with zinc. Doxycycline (Dox) was bound non-covalently into the membranes by base-acid interactions between amino groups of Dox and carboxyl groups of the membranes. To control the functionalization degree of the adsorption, isotherms of Zn^2+^ and Dox on the HOOC-Si-membrane were previously studied [10]. For the Zn^2+^ and Dox functionalization HOOC-Si-membranes were incubated at room temperature and under continuous shaking in aqueous solutions (pH = 7) of 330 [ZnCl_2_]_0_ mgL^−1^ for 60 min or 800 [Dox]_0_ mgL^−1^ for 30 min, respectively. The attained zinc adsorption was 3 μg Zn/mg membrane and the Dox adsorption was 76.2 μg Dox/mg membrane [10]. Three different membranes were tested, (1) SiO_2_-NPs doped membrane (HOOC-Si-Membrane), (2) SiO_2_-NPs doped membrane functionalized with Zn (Zn-HOOC-Si-Membrane) and (3) SiO_2_-NPs doped membrane functionalized with Dox (Dox-HOOC-Si-Membrane).

### 2.2. Animal Experimentation

For this study, six New Zealand–breed experimentation white rabbits with identical characteristics (weight 3.5–4 kg, age 6 months) were selected. Rabbits were adequately sheltered; Rabbit maintenance Harlan-Teckland Lab Animal Diets (2030) provided daily ad libitum food and water. The experiment was developed following the US National Institute of Health (NIH for Care and Use of Laboratory Animals) and European Directive 86/609/EEC guidelines concerning animals care and use for experimentation. This study also fulfilled the European Directive 2010/63/EU about the animals protection for scientific purposes and with all local laws and regulations. The Ethics Committee of the Institution (CCMI-Ref 026/18) approval was obtained. The minimum number of animals was used for ethical reasons as required by the legislative framework. Concerning animal experimentation and histological methods, comparable models have been published [6].

### 2.3. Surgical Procedure

Before the surgery was realized, vital signs were obtained from each rabbit and then they were immobilized. Propofol (5 mg/kg) and Midazolam (0.25 mg/kg) were employed as anesthetics and infiltrated intravenously for induction along with 2.8% inspired sevoflurane gas inhalation. Ketorolac (1.5 mg/kg) and tramadol (3 mg/kg) provided the analgesia. Once rabbits were sedated and prepared, incisions of Number 15 scalpel blade between their eyes and their ears bases were made. After connecting in the skull midline the two incisions with another one, a surgical triangular field was done. The connective, muscular, and epithelial tissues were separated from the operation field with a Prichard periosteotome and a sterile saline solution was applied to wash the skull surface. On the parietal bone, on each side of the skull midline, 3 mm apart, four critical bone defects (diameter 8 mm, depth 3 mm) were done using a trephine (Helmut-Zepf Medical Gmbh, Seitingen, Germany) coupled to an implant micromotor operating at 2000 rpm under saline irrigation. The trephine showed an external diameter of 8 mm, a length of 30 mm, and teeth of 2.35 mm. In order to remove the inner table and the medullary bone in every defect, piezosurgery was handled. A periodontal probe was used to control the depth. To cover each of the three bone defects, a randomly allocated membrane was used. The fourth bone defect was not covered (unloaded). A specific software (Research Randomizer, V. 4.0, Urbaniak GC and Plous S, 2013) was generated to randomize the sequence. Tissucol (Baxter, Hyland S.A. Immuno, Rochester, MI, USA), a fibrin tissue adhesive, was applied to fix the membranes, and it was placed on the bone rims adjacent to the defects. Once the flaps were moved back to their initial positions, limited mobility and proper adhesion of these membranes were confirmed. Using resorbable material, sutures were made on the following sequences: Periosteal (4/0), sub-epidermal (4/0), and skin (2/0). Simple stitches were applied as close as possible to the edge. In order to clean the wound, a sterile saline solution was applied. Anti-inflammatory analgesia (carprofen 1 mL/12.5 kg and buprenorphine 0.05 mg/kg) was administered. Six weeks after surgery, the animals were sacrificed with a potassium chloride solution intravenous overdose. After the procedure, the obtained tissue samples were cut and separated individually [8].

### 2.4. Micro Computed Tomography (Micro-CT)

After the brain mass was extracted and the skull was washed with a sterile saline solution, rabbit skulls were assessed by Computerized Tomography employing a Bruker Albira preclinical CT scanner. The highest quality available was employed for this analysis; 1000 image 360° radiographic projection at 45 kV, and a 30-min acquisition time. Albira Suite software and standard reconstruction settings were used for the tomographic reconstructions [17], generating 2D and 3D volumes with a resolution of 8.3 voxels/mm. Average bone density in Houndsfield Units (HU), total average volume × 1000, and hot average values were calculated. PMOD-Biomedical Image Quantification software (PMOD technologies LLC), was used by positioning 2-mm spherical volumes of interest (miniVOIs) within each lesion in a rosette arrangement, to measure the bone just formed. Albira Suite software was used to obtain high resolution reconstructions of a 10 mm^3^ volume within each lesion, generating volumes with a resolution of 20-voxels/mm (Figure S1). *BoneJ* [18], a free plugin for *ImageJ* [19], was used to evaluate bone architecture. To automatically perform the analysis on all sub-volumes, an *ImageJ* script was created using the same HU density threshold and *BoneJ* settings using crop (central lesion region) and threshold (changes in bone density). Analysis v5 [high crop (300px) and low threshold (500)] was selected. It showed the biggest differences between lesions, as lower threshold offered more sensitivity to smaller changes in bone density. Trabecular thickness, which calculates the breadth of foreground and background to provide trabecular width and separation, was assessed. Connectivity (Euler, Δ(χ), net connectivity, and connectivity density) was also measured, as trabecular bone can be treated as a network. Its connectivity density was obtained by dividing the estimated connectivity by the sample volume. To describe the trabecular structure complexity, the skeleton analysis was registered as counts and measure branches and junctions of a bone structure image.

### 2.5. Histological Processing of Samples

From each rabbit skull, samples were obtained cutting them in an anatomical sagittal plane. A 5% buffered formaldehyde solution (pH 7.4) was employed to fix the undecalcified bone. An oscillating autopsy saw (Exakt, Kulzer, Wehrheim, Germany) was used to retrieve blocks from the regenerated bone defect. Subsequently, the dissected specimens were immersed in 4% formaldehyde and 1% calcium solution included in acrylic resin and prepared for ground sectioning. To visualize the mineralized bone, von Kossa (VK) silver nitrate stain (Sigma-Aldrich Chemical Co., Poole, UK) was applied (scale bar, 850 μm). An Olympus SZ-CTV stereomicroscope (Olympus, Tokyo, Japan) with 1.2× lenses was employed to study bone VK morphometric study. A digital signal processor (DSP) 5050Zoom camera (Olympus, Tokyo, Japan) took the pictures. For each bone defect, one image was obtained. The following structural indexes were measured: Bone surface (BS), percentage of bone area [BS/total surface (TS)], bone perimeter (BPm), and bone thickness (BTh) (scale bars, 1000 μm). A metachromatic dye was used for rapid contrast tissue analysis and histological staining (Merck Toluidine Blue-Merck, Darmstadt, Germany). This was attained with a 1% toluidine blue (TB) solution with a pH of 3.6 and it was adjusted with HCl. For 10 min at room temperature (23.0 ± 1.0 °C), samples were exposed to the dye with distilled water, and air dried. To observe calcein deposition into the newly deposited bone matrix, fluorescence images were also obtained. An Eclipse LV100 microscope (Nikon, Tokyo, Japan) with 20× and 5× lenses was employed to carry out toluidine and fluorescence morphometric bone studies, respectively. A DSPDS-Fi1 camera (Nikon, Tokyo, Japan) along with a NISElements BR 4.0 software (Nikon, Tokyo, Japan) took the pictures. Osteocytes, osteoblasts, osteoclasts, blood vessels and macrophages (M1 and M2 and ratio M1/M2) were assessed at TB images. The M1 and M2 macrophages number and the ratio M1/M2 were analyzed with morphology criteria by coloration with toluidine blue. They exhibit a distinctive morphology, with round, vacuolized or fried egg-shaped [20] for M1, or elongated, spindle-shape or fibroblast-like appearance for M2 [12] (scale bars, 10, 50 and 100 μm). Image analyses were realized using *ImageJ* software. In each bone defect, four images were taken and analyzed. At fluorescence, one image was obtained for defect and total area (TA), osteoid area (OA), the percentage of the total area occupied by osteoid (OA/TA), perimeter of the osteoid (OPm), the area occupied by mineralized bone (BA), its percentage respect to the total area of the defect (BA/TA), as well as its perimeter (BPm) were registered (scale bar, 1000 μm).

### 2.6. Statistical Analysis

Means and standard deviations (SD) were obtained. Nonparametrical Friedman test was used for variance analysis and non-parametric pairwise comparison of Friedman rank sums method for post-hoc analysis was employed. Level of significance was set at *p* ≤ 0.05. Assessment was undertaken by means of IBM SPSS Statistics v.24 software package.

## 3. Results

*BoneJ* analysis showed that HOOC-Si-Membranes produced more maximum branch length (MXBRLE), total volume (TV) and trabecular thickness maximum (TBTHMX) than Dox-HOOC-Si-Membranes. Zn-HOOC-Si-Membranes induced more MXBRLE and TV than Dox-HOOC-Si-Membranes, and Dox-HOOC-Si-Membranes showed more average branch length (ABRLE) than the control group (Figure S2) (Table S1). Micro-computed tomography evaluation of both the centre of the defect (crop) and bone structures density (threshold) were able to distinguish differences among biomaterials. A representative Two-dimensional (2D) Micro-CT image from a HOOC-Si-Membrane and Zn-HOOC-Si-Membrane groups are provided in Figures S3 and S4, which showed partial or complete images of bony bridging formations, respectively. Two-dimensional Micro-CT image from a Dox-HOOC-Si-Membrane and control groups are shown in Figures S5 and S6, which portray scarce new bone formation at the defect centre in both groups. *BoneJ* analysis showed that both Zn-HOOC-Si and HOOC-Si-Membranes produced higher maximum branch length and total volume than Dox-HOOC-Si-Membrane. Dox-HOOC-Si-Membrane produced higher average branch length and total volume than in control group (*p* < 0.05) (Table S1). Hunsfield density from total miniVOIs analysis showed that samples treated with Dox-HOOC-Si-Membranes induced lower normalized density and total average volume than the rest of the groups (*p* < 0.05), which showed not statistical differences among them. Animals treated with Dox-HOOC-Si-Membranes attained lower hot average than Zn-HOOC-Si-Membranes (*p* < 0.05), which did not show differences when compared with HOOC-Si-Membranes and the control group (Tables S2 and S3). When the central miniVOIs hot average density was analysed, specimens treated with Dox-HOOC-Si-Membranes showed lower values than the control group, which did not differentiae when it was compared with HOOC-Si-Membranes or Zn-HOOC-Si-Membranes. Normalized density and total average volume values were similar among groups in the central miniVOIs analysis (*p* < 0.05) (Tables S4 and S5). The periphery miniVOIs analysis unveiled that animals treated with Dox-HOOC-Si-Membranes showed lower normalized density and hot average than the rest of the groups (*p* < 0.05), without differences among them. The total average volume in periphery analysis was lower in samples treated with Dox-HOOC-Si-Membranes than in the Zn-HOOC-Si-Membranes group, which did not show significant differences with respect to groups treated with HOOC-Si-Membranes and the control group (Tables S6 and S7).

**Figure 1:**
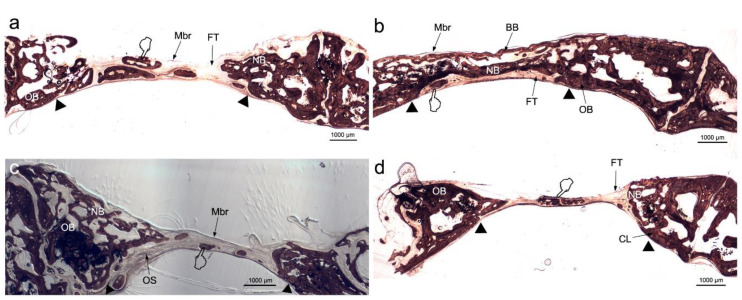
Bone histomorphometry obtained by dye with von Kossa silver nitrate stain, to visualize formed mineralized bone, at six weeks of follow up, around HOOC-Si-Membranes (**a**), Zn-HOOC-Si-Membranes (**b**), Dox-HOOC-Si-Membranes (**c**), and blank control (no membrane) (**d**). The presence of interstitial connective or fibrous tissue was evidenced in some instances. Trabecular bone formation were observed along the margin of calvarial defect (arrow head), and within the defect. **BB**, bony bridging; **FT**, fibrous tissue; **Mbr**, membrane; **NB**, new bone; **OS**, osteoid; **OB**, old bone, cement line (**CL**). Pointers mark scattered bone islands, indicating with new bone.

All membranes-treated bone defects attained higher bone perimeter (BPm) than the control group (Table 1 and Table 2) (Figure 1), as observed by the Von Kossa (VK) stain. The samples treated with Zn-HOOC-Si-Membranes and Dox-HOOC-Si-Membranes showed higher amount of new bone, i.e., ratio bone surface/total surface (BS/TS) than the control group (Figure 1b–d). Animals treated with Zn-HOOC-Si-Membranes attained higher BS/TS than those treated with HOOC-Si-Membranes. Intersticial connective tissue and areas of trabecular bone formation were visible in all samples (Figure 1). Adipocyte-like surrounding tissues were also shown, particularly in the control group. The control group bone defect was filled with connective tissue and a few immature bone trabeculae (Figure 1d). In Zn-HOOC-Si-Membranes were observed bony bridging processes (Figure 1b). In all groups, except when Zn-HOOC-Si-Membranes were used, bone only regenerated at the defect edge, without evidence of bridging, and a decrease was present in the defect area (Figure 1a,c,d).

**Figure 2:**
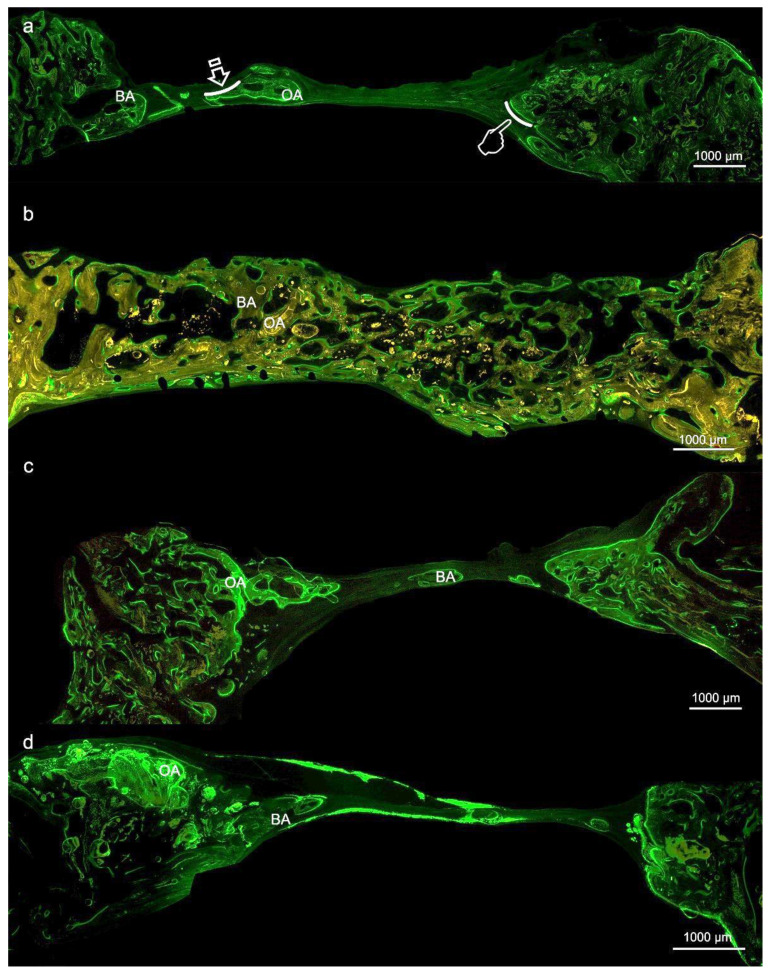
Bone histology obtained by fluorescence with calcein at the region of interest to see mineralized bone, at six weeks of healing time after using HOOC-Si (**a**), Zn-HOOC-Si (**b**), Dox-HOOC-Si; (**c**) loaded membranes, and control (no membrane) (**d**). Arrows indicate bone perimeter (**BP**). Pointers indicate osteoid perimeter (**OP**). **BA**, bone area; **OA**, osteoid area.

At the histological figures obtained with fluorescence calcein, membrane-treated bone defects (Figure 2a–c) showed similar new bone formation, i.e., bone area respect total area (BA/TA), with higher values (*p* < 0.05), than the control group. Samples treated with Zn-HOOC-Si-Membranes attained higher values of the osteoid area respect to the total area (OA/TA) than in the control group (*p* < 0.05) (Figure 2d), but differences among the groups of animals treated with membranes were not significant (*p* > 0.05). Specimens treated with membranes promoted higher bone perimeter (BPm) than the control group (*p* < 0.05) (Table 3 and Table 4) (Figure 2).

**Figure 3:**
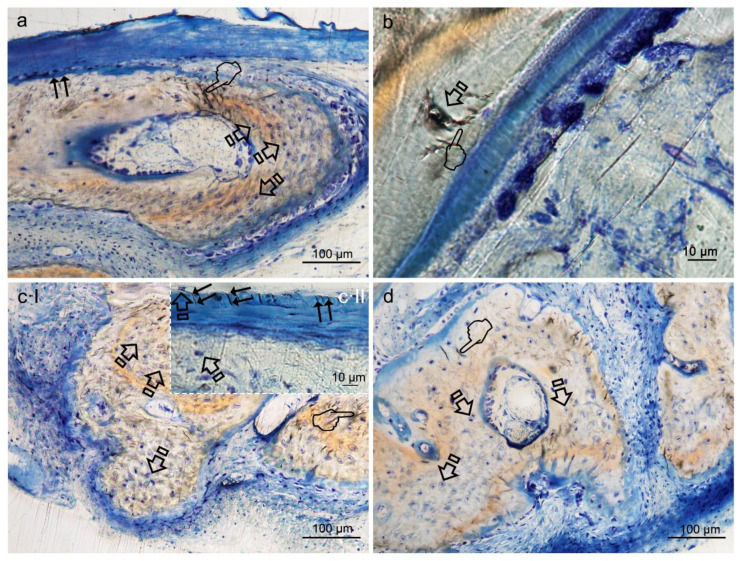
Bone histology obtained after using HOOC-Si (**a**), Zn-HOOC-Si (**b**), Dox-HOOC-Si (**c**) loaded membranes, and blank control (no membrane) (**d**), dyed with toluidine blue to visualize mineralized bone, at 6 weeks of healing time. Single arrows point the presence of osteocytes. Canaliculi crossed through the bone matrix to bridge up a large extend of the membranes (double arrows) (Figure 3a,(cII)). The extended network of osteocyte dendritic processes which reproduced the characteristic dendritic morphology joined at gap junctions (pointers) (Figure 3a,b,(cI),d). They were connected and intersected with each other by fine cytoplasmic processes and their branches. At high magnification, the osteocyte appeared embedded within its mineral lacuna (Figure 3b,(cII)).

Histological images obtained with toluidine blue (TB), allowed to detect that the calvarial defects treated with any of the tested membranes attained higher number (or counting) of osteocytes than the uncovered defect (*p* < 0.05) (Table 5 and Table 6) (Figure 3a–d).

The newly-formed bone at the bone defect showed viable bone and large osteocytes lacunae bridging up the greatest part of the membranes. Interconnectivity between osteocyte canaliculi in old and new bone was observed. The trend was as follows: HOOC-Si-Membranes = Zn-HOOC-Si-Membranes = Dox-HOOC-Si-Membranes > Control group. Counting of osteoblasts was higher when Zn-HOOC-Si-Membranes or Dox-HOOC-Si-Membranes were used and compared with the control group (*p* < 0.05) (Table 5 and Table 6). The trend was as follows: Dox-HOOC-Si-Membranes ≥ Zn-HOOC-Si-Membranes ≥ HOOC-Si-Membranes ≥ Control group. 

**Figure 4:**
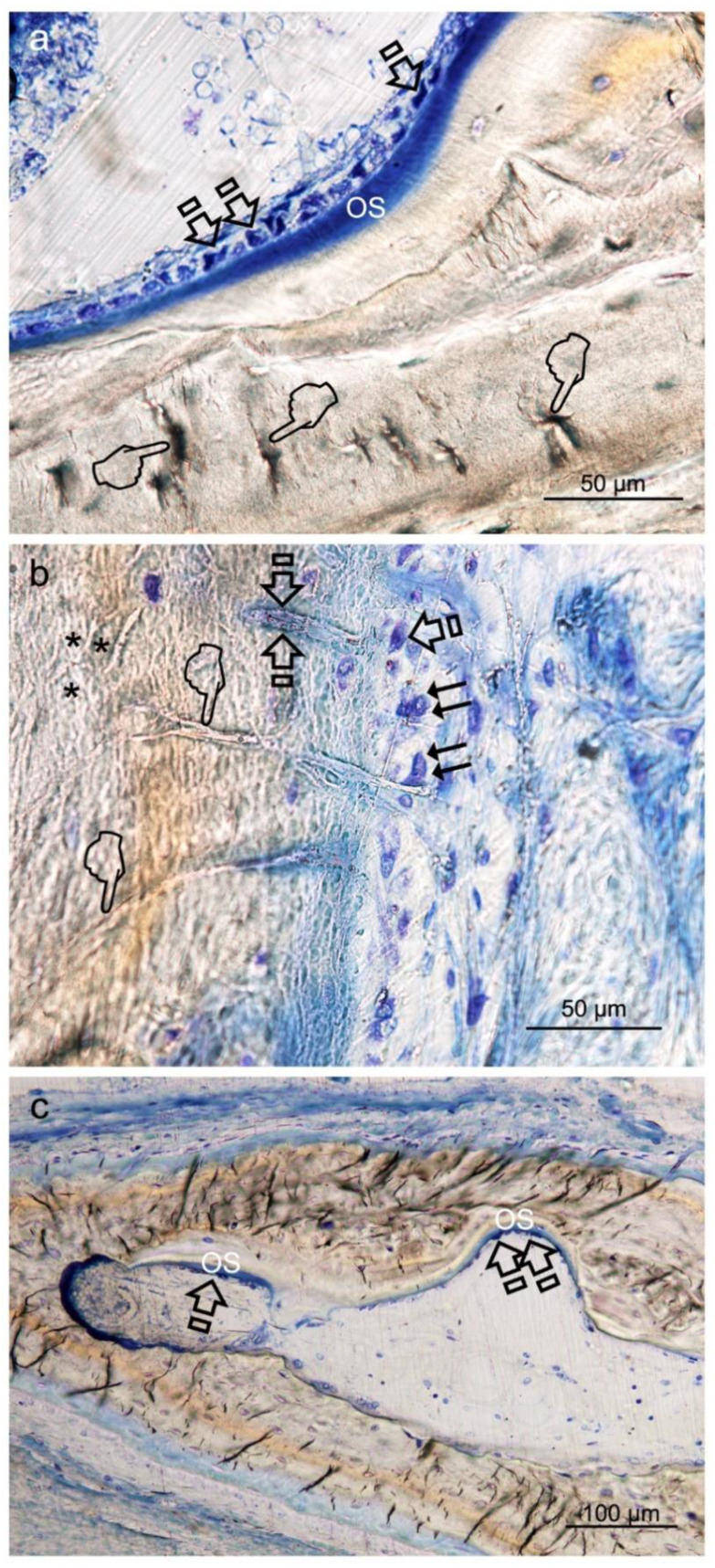
Bone histology images obtained after using Zn-HOOC-Si (**a**), Dox-HOOC-Si (**b**) loaded membranes, and control (no membrane) (**c**), by coloration with toluidine blue to visualize mineralized bone, at 6 weeks of healing time. Single arrows point the presence of aligned osteoblasts, with typical cuboid shape (Figure 4a). Polarized osteoblast (Figure 4a,b) show part of the cell membrane in direct contact with the bone surface, unveiling many cytoplasmic processes, that achieve the newly deposited osteoid (**OS**). Double arrows are indicative of osteoclasts; pointers mean canaliculi and faced arrows, an osteocyte. Asterisks are located at zones of gap junctions (Figure 4b).

Osteoblasts showed a strongly basophilic cytoplasm, indicating the ability to produce a large amount of extracellular matrix (Figure 4a,b).

**Figure 5:**
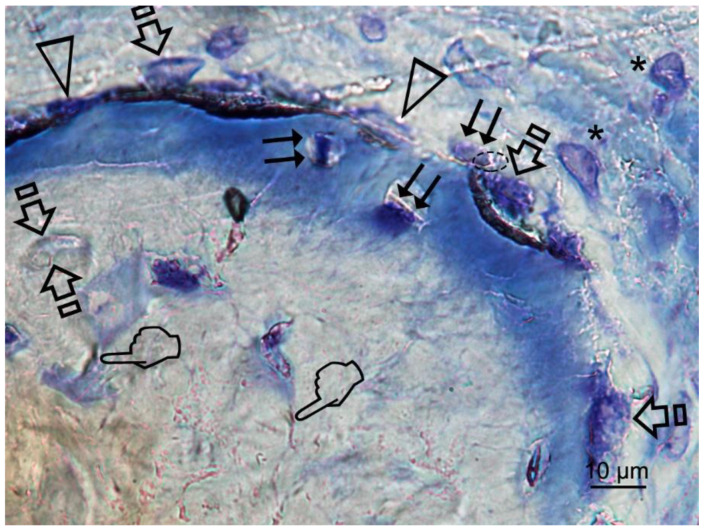
Bone histology images obtained after using Dox-HOOC-Si-membrane, by dye with toluidine blue to visualize mineralized bone, at six weeks of healing time. Single arrows point to the presence of osteoclasts. As opposed to the contact surface of the bone through the ruffled border forming a sealing zone (podosome), the functional secretory domain can be adverted at the peak of the single arrows. Osteoclasts and osteoblasts connect with each other through direct cell-cell contact and extracellular matrix interaction (dotted circle). Double arrows indicate osteoblasts, and pointers mean canaliculi and faced arrows, osteocyte. Asterisks are located close by some macrophages. Lining cells, in close contact with osteoclasts, are signaled by arrow head.

The control group attained the highest counting of osteoclasts. Samples treated with Dox-HOOC-Si-Membranes achieved the lowest number of osteoclasts, significantly different from the control group (*p* < 0.05) (Table 5 and Table 6). The trend was as follows: Dox-HOOC-Si-Membranes < Control ≥ HOOC-Si-Membranes = Zn-HOOC-Si-Membranes. A fitted junction between the bone surface and the basal membrane of the osteoclasts formed a sealed compartment known as the acting ring which includes the characteristic ruffled border, as a part of an external vacuole (Figure 5). 

**Figure 6:**
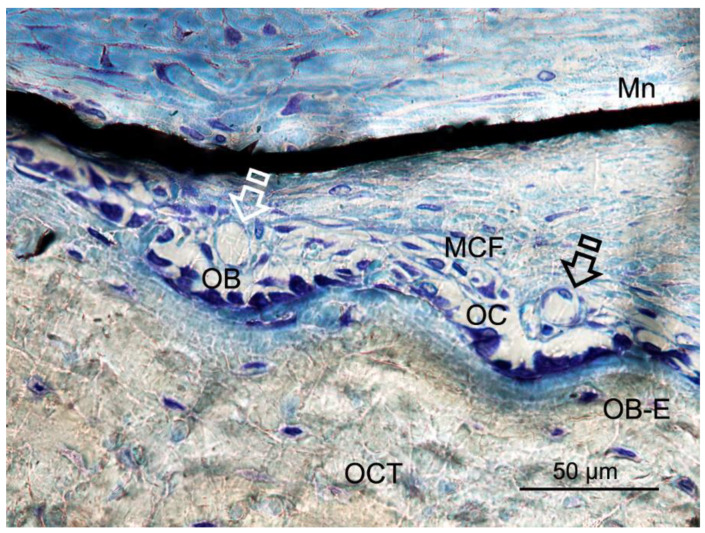
Bone histology images obtained after using Zn-HOOC-Si loaded membrane (**Mn**), by dye with toluidine blue to visualize mineralized bone, at six weeks of healing time. Single arrows point the presence of blood vessels; both osteoblasts and osteoclasts are in close contact with marrow elements and the contiguous vasculature. Osteocytes (**OCT**), osteoblasts (**OB**), entrapped osteoblasts (**OB-E**), osteoclasts (**OC**) and macrophages (**MCF**) may be observed.

Zn-HOOC-Si-Membranes produced the highest number of blood vessels among groups. Significant differences did not appear in the counterparts (*p* < 0.05) (Table 5 and Table 6). The trend was as follows: Zn-HOOC-Si-Membranes > HOOC-Si-Membranes = Dox-HOOC-Si-Membranes = Control. Blood vessels appeared in close vicinity to cells groups (Figure 6).

**Figure 7:**
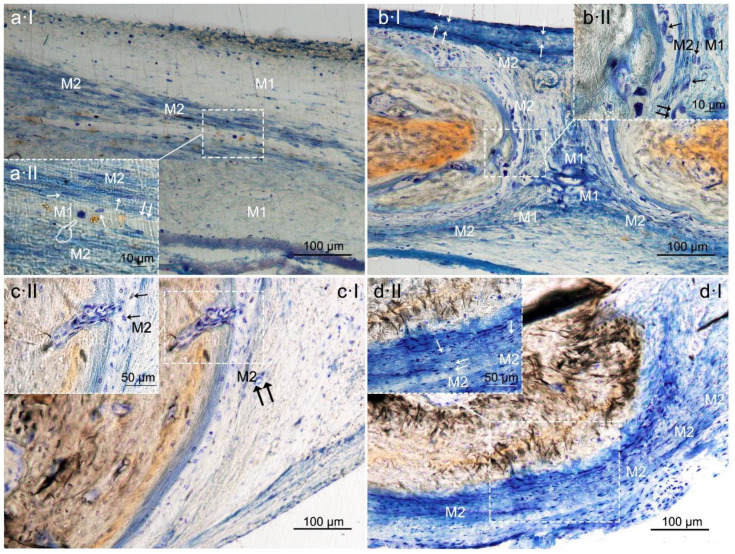
Bone histology images obtained after using HOOC-Si (**a**), Zn-HOOC-Si (**b**), Dox-HOOC-Si (**c**) loaded membranes, and blank control (no membrane) (**d**), by dye with toluidine blue to visualize macrophages at the bone defect, at 6 weeks of healing time. M1 (pointers) and M2 (single arrows) macrophages were observable at the centre of the defect (Figure 7a). M1 showed increase size and granularity, nearly round or irregularly spherical shape, with more lamellipodia. M2 exhibited elongated or spindle-shaped appearance and long filopodia, with a loss of nuclear volume and induction of chromatin condensation (Figure 7a–d). Small vessels are also shown (double arrows). Cells have invaded the experimental tested membrane (faced arrows) (Figure 7(bI)).

After statistical analysis, it was shown that the samples treated with both HOOC-Si-Membranes (Figure 7a) and control group induced higher number of M1 macrophage phenotype cells than bone defects treated with Zn-HOOC-Si-Membranes. M1 macrophages were also more numerous in the control group than in animals treated with Dox-HOOC-Si-Membranes or Zn-HOOC-Si-Membranes. M2 were lower in the control group (Figure 7d) than in cases treated with any of the tested membranes (*p* < 0.05) (Figure 7a–c). Groups treated with both HOOC-Si-Membranes and the control group showed the highest ratio M1/M2, and the lowest ratio M1/M2 was observed in animals treated with both Dox-HOOC-Si and Zn-HOOC-Si-Membranes (Table 7 and Table 8).

## 4. Discussion

Based on morphological criteria, the low ratio pro-inflammatory M1/anti-inflammatory M2 macrophages has promoted a pro-healing phenotype, and induced bone regeneration and vascularization in rabbit bone defects, treated with Zn-HOOC-Si polymeric nanostructured membranes. In the guided bone regeneration application, Zn-HOOC-Si-Membranes have served as a barrier to prevent epithelial cells and connective tissue migration, while maintaining a space to allow bone ingrowth. Zn-HOOC-Si-Membranes also produced higher maximum branch length and total volume than Dox-HOOC-Si-Membranes. In the absence of any membrane covering the bone defect (control group), the bone regeneration was in general terms lower than in the rest of the groups, as both new bone (BS/TS, BA/TA) and bone perimeter (BPm) attained the lowest values in the control group when both, von Kossa and the fluorescence techniques (Figure 1d and Figure 2d) were used for the assessment (Table 1, Table 2, Table 3 and Table 4). Furthermore, micro-CT indicated little formation of new bone in the bone defect area of the control group. This confirmed that the prepared bone defect model can be used as a guide for bone regeneration within the time of study [21]. Moreover, first null hypothesis is rejected. The rise in these structural indexes, in comparison with those obtained in the control group, was interpreted as the replacement of older with more resilient and younger bone (Figure 1), as these are crucial requirements for bone remodeling, necessary to repair damaged bone [22]. 

Osteocytes play a key role in the formation and remodeling of bone [23]. They are mechano-transducer cells and crucial determinants of bone quality [23]. Certain osteocyte morphologic parameters, such as number, size, alignment with respect to bone lamellae, density, vicinity to small and large blood vessels, lacuna-canalicular interconnectivity between neighboring and distant osteocytes, and quantity of them reveal vital clues, regarding the bone quality status [23]. The present analysis showed higher counting of these cells in all animals treated with the experimental, regardless the type of biomaterial (Table 5 and Table 6) (Figure 3a,c,d). Therefore, it can be inferred that the present membranes reinforced the role of osteocytes to; (i) function as bone remodeling central orchestrators that can integrate to regulate bone mass both, hormonal and mechanical signals, (ii) induce new recruiting bone formation at sited of damage by inducting mesenchymal stem cells, and (iii) potentially play a role in calcium and phosphate mineral homeostasis, as previously stated [24].

Osteocytes are important determinants of bone quality, and locally modulate osteoblasts and osteoclasts activity [25]. Zn-HOOC-Si-Membranes and Dox-HOOC-Si-Membranes produced higher amount of osteoblasts than the control group and. Therefore, animals treated with HOOC-Si-Membranes showed similar number of osteoblast than the control group (Table 5 and Table 6). As a result, it might be inferred that Zn-HOOC-Si-Membranes and Dox-HOOC-Si-Membranes equally stimulate the mesenchymal stem cells, which are pluripotent cells with the potential to differentiate into osteoblasts, enhancing advanced bone regeneration for treating critical bone defects. The osteoblasts have also been shown to maintain bone ossification and bone repair [26]. Osteoblast lineage cells function as a reservoir of phosphorous and calcium. They produce osteocalcin, alkaline phosphatase and type I collagen, known as osteoid when first deposited and not yet mineralized [27]. The osteoid was observed as a homogeneous fringe in a clear blue color between the aligned osteoblasts and the mature bone (Figure 4a,c). Both osteoid area (OA) and osteoid perimeter (OPm) were significantly higher when Zn-HOOC-Si-Membranes were used and compared with the counterparts (Table 5 and Table 6) (Figure 2). Furthermore, Zn-HOOC-Si-Membranes promoted the highest new bone formation (BS/TS) among groups (Table 1) and induced a robust bridging process joining the edges of the defect, in close contact with the membrane (Figure 1b and Figure 2b). Even more, Zn-HOOC-Si-Membranes provided a similar architecture to that one of the native calvarial bone (Figure 1b) and, in this group, the new bone almost filled the defect area (Figure S4). The dendritic morphology of osteocytes might have contributed to enlarging the surface area beneath the Zn-HOOC-Si-Membranes (Figure 2b). These osteocytes’ filapodia serve to sense extracellular stimuli like hormones, cytokines and mechanical loading [28]. This finding has been identified as a sign of young bone formation [29], and confirm that tissue regeneration takes place in the surrounding area of, and in response to, preferentially certain implanted biomaterials [23]. The carboxylate groups might have functioned as cell binding sites [30], probably after Zn^++^ release.

Once formed, bone undergoes remodeling with the key participant being the osteoclast [31], that is a major stimulus for bone formation [32]. The resorption and formation of bone is stable at equilibrated physiological conditions. Osteoclasts and osteoblasts can communicate through direct cell to cell contact, as may be observed at Figure 5, cytokines and extracellular matrix interacts with each other, as previously reported [33]. Nevertheless, when there is an imbalance, bone function or architecture will be abnormal [33]. As observed in the present research, a decrease of osteoclasts after treating the bone defect with Dox-HOOC-Si-Membranes was linked to an increase of osteoblasts (Table 5 and Table 6), and a rise of new bone (BS/TS, BA/TA), when compared with the control group (Table 1, Table 2, Table 3 and Table 4). Bone formation and osteoid induction in alveolar bone after doxycycline administration have been reported [34]. To avoid for intracellular alkalinization and thus hyperpolarization, osteoclasts generate chlorhydric acid, creating a pH of about 4.5. This can trigger the mineralized components degradation, and result in the exposition of the organic matrix, which can lead to the degradation of its main component (type I collagen) by collagenases [35]. Therefore, a reduction in the number of osteoclasts leads to a lower demineralization, increase bone formation and inhibition of the collagenolitic activity, due to the presence of doxycycline [36], favoring bone remineralization [37]. All these described events will explain the attained increases at both new bone formation and bone perimeter at the analyzed defects (Table 2 and Table 4). Even more, the Dox-HOOC-Si-Membranes suppressed osteoclasts activity, which otherwise causes bone degradation [38]. The lower osteocyte count, in specimens that were not treated with any membrane (control group), could account for the higher number of osteoclasts, attained in the control group when compared with the other groups (Table 5), as expected following other publications [24,39]. Control group showed little bone healing (Figure S6).

Bone formation regulating and remodeling do not occur without proper blood supply and vascularization [38]. In the absence of any membrane as it does occur in the control group, the spontaneous vascular ingrowth speed from the existing blood vessels is normally several tenths of micrometer per day, which is too slow to afford adequate nutrient to the cells in the interior of the defect. Therefore, supplementary strategies for stimulating angiogenesis are vital to ensure the large tissue-engineered construct survival [40]. The highest vasculature was shown after using Zn-HOOC-Si-Membranes (Table 5 and Table 6), which showed clear signs of remodeling (Figure S4), based on both recruitment of cells and the supply of nutrients, as previously stated [41]. Angiogenesis always precedes osteogenesis [42]. Thereby, it can be suggested that Zn-doping can facilitate higher bone healing regenerative efficiency. In newly formed bone, vascular morphogenetic proteins, especially angiopoietins and vascular endothelial growth factor (VEGFs), regulate neovasculature/neoangiogenesis [38]. Osteoblasts, more abundant in samples treated with Zn-HOOC-Si-Membranes and Dox-HOOC-Si-Membranes (Table 5 and Table 6), secret VEGFs and endothelial cells secret bone morphogenetic protein (BMP)2 to combine osteogenesis and angiogenesis [43]. Only cells that are 100–200 µm from blood vessels can receive oxygen through diffusion [4], as it may be observed at Figure 6. Beyond this distance, blood vessels by sprouting can arise as a response to local cues derived from hypoxic tissues. There are two events, which underlie sprouting angiogenesis, growth and stabilization. In the growth phase, the existing vessels vasodilatation leads to enhance basement membrane permeability and degradation mediated by metalloproteinases (MMPs) release [4]. Both Doxycycline and Zn are potent MMPs inhibitors [36], but Zn particularly does also stimulate the osteogenic and angiogenic stem cells differentiation [44]. It is speculated that this potential could offset the inhibitory capacity of MMPs. 

Zn-HOOC-Si-Membranes are preferred, as silica and zinc together, not only displayed osteoconductivity and new bone formation (Table 1 and Table 2), but neovascularization (Table 1, Table 2, Table 5 and Table 6), and positively influenced macrophages polarization (Table 7 and Table 8). Among the animals treated with membranes, bone defects covered with HOOC-Si-Membranes attained the highest pro-inflammatory M1 counts and the lowest anti-inflammatory M2 counts (Figure 7a), and Zn-HOOC-Si-Membranes induced the opposite performance (Table 7 and Table 8) (Figure 7b). To obtain anequilibrium between osteointegration and osteoimmunology, several approaches have been implemented, in order to obtain modifications in biomaterials that could modulate the associated immunological reactions. It has been established that engineered modified biomaterials can generate an osteogenic immune microenvironment [43]. Indeed, by the end of the experiment, a complete bone bridge was not formed in any case (Figure 1a and Figure 2a); only a discontinuous bony regeneration attempt was observed parallel to the membrane, attempting to bridge both sides of the defect (Figure S3). The new bone formed mainly at the bottom and peripheral regions of the defect (Figure 1a,b), which is typical of an early time point in neoformation has been reported previously [32]. Incorporation of silicon has been shown to enhance the osteoblast-like cell activity and bioactivity, but at the expense of poorly crystallized apatite layer, which leads to an increase in resorbability and new bone dissolution [45]. M1 are crucial in damaged tissue reorganization via the activation of a variety of enzymes and immunostimulatory cytokines (TNFάIL-1β and IL-6) [20], leading to enhance inflammation, tissue injury and fibrosis [12]. In the light of the obtained results, it is speculated that specimens treated with both HOOC-Si-Membranes and control group with higher M1/M2 ratios (Table 7 and Table 8) (Figure 7a,d) will follow a chronic pro-inflammatory tissue reaction, leading to negative consequences for tissue remodeling, such as fibrous encapsulation, as stated previously [46]. At the end of the study, some control defects, as well as a certain amount of HOOC-Si-Membranes samples were completely filled with fibrous or adipose-like tissue, which is a characteristic of immature tissue [6,47], and quite a few newly formed, island-like bond (Figure 1a,d and Figure 2a,d). The presence of fibrous tissue complies with the recruitment of inflammatory cells and presence of inflammatory microenvironment [43]. Zn-HOOC-Si-Membranes and Dox-HOOC-Si-Membranes (Figure 7c) produced the lowest M1 counts (Table 7 and Table 8). M1 produces matrix metaloproteinases (MMPs) that diminish when the M1 population decreases. Zinc and doxycycline are MMPs inhibitors [36] and, thereby, their pro-healing role is jeopardized. M2 counts, higher in samples treated with both, Zn-HOOC-Si-Membranes and Dox-HOOC-Si-Membranes, have been shown to promote angiogenesis and vascular/matrix remodeling [48], which corresponded with the highest vasculature attained among groups in the present research (Table 5 and Table 6). Additionally, M2 cells mainly participate in tissue remodeling and immunoregulation processes [12], decreased phagocytic capability [11], and strongly reduced the oxidative stress damage after using zinc and doxycycline [49]. The increase in the number of M2 macrophages leads to an immunological response shift through the production of anti-inflammatory cytokines and inhibition of reactive oxygen species [20]. Both factors induce the polarization of M1 to M2 subtype [20], via an IL-4-dependent pathway [50], suggesting the immunomodulatory capability of zinc [43]. This modulation resulted in low thickness or not fibrotic capsule development around the Zn-HOOC-Si nanostructured membranes (Figure 1b and Figure 2b). Madden et al. (2010) [51] also fabricated poly (2-hydroxyethyl methacrylate-comethacrylic acid) (pHEMA-co-MAA) hydrogel scaffolds and obtained maximum vascularization, minimal fibrotic response that combined with an increase number of M2 phenotype macrophage cells.

It was hypothesized if differences in chemical composition of the membranes would modulate macrophages phenotype ratio (M1/M2). The investigation and further understanding of the mechanisms that lead to the polarization of the macrophages and the switch between M1andM2 states are of current interest and might contribute to novel therapeutic approaches [52]. Macrophages morphology, and shape are important factors for the modulation of macrophages phenotypic polarization [53,54]. Since pro-inflammatory M1 and anti-inflammatory M2 macrophages denote two poles of a continuum of overlapping cellular activities; the morphological parameters were used to define the polarization outcomes, as previously reported [12]. M2 showed elongated shape and long filopodia. M1 were nearly round or irregularly spherical with more lamellipodia [53]; an example is visible at Figure 7. The ratio M1/M2 achieved the lowest values among groups when Zn-HOOC-Si-Membranes and Dox-HOOC-Si-Membranes (Table 7 and Table 8). Therefore, the second null hypothesis must be rejected as our study attempted to elucidate further application of Zn in immunomodulation, and this has been confirmed. Zinc is an essential trace element and is part of some key enzymes and transcription factors. It has been identified as a crucial element for the immune system development. The presence of zinc can result in an enhancement of anti-inflammatory cytokines expression and the maintenance of an anti-inflammatory environment [43]. Specifically, zinc can induce the differentiation of monocytes to macrophagesand can increase pro-inflammatory cytokines release such as IL-1, IL-6, and TNF-α [43]. Moreover, the attachment and spread of macrophages may be facilitated by the porous structure; then, the physical and mechanical signals of porous surface, about 6.99 μm in Zn-HOOC-Si-Membranes [10] could be translated into biological signals, and subsequently modulate local microenvironment and macrophage polarization [43]. 

Nevertheless, the lower values of bone density obtained in samples treated with Dox-HOOC-Si-Membranes, in comparison with those attained by Zn-HOOC-Si-Membranes (Tables S1, S3 and S5), correspond with data obtained in the histomorphometric assessment from Von Kossa and calcein staining analysis (Table 1, Table 2, Table 3 and Table 4). Zn-HOOC-Si-Membranes promoted more bone perimeter and vasculature than Dox-HOOC-Si-Membranes (Table 4 and Table 6). An insufficient vascularity of the bone can result in a decrease of bone formation [42]. Nevertheless, it has been previously shown that doxycycline facilitates an increase in trabecular bone area and length of the trabecular surface covered by osteoid [55], though the Micro-CT analysis, performed in the present research inferred lower values in samples, treated with Dox-HOOC-Si-Membranes than those treated with Zn-HOOC-Si-Membranes. Moreover, maximum branch length and total volume attained minor counts in doxycycline-loaded membranes (Table S1). Hence, new bones in animals, treated with Zn-HOOC-Si-Membranes-treated animals, was formed by multiple interconnected ossified trabeculae and was shown in close contact with the membrane surface (Figure S4c,d). It may be taken as a sign of successful bone conduction and a robust bony bridging, as published previously [56]. Nevertheless, new formed bone was not continuous in some of the scanned sections (Figure S4a), indicating the presence of reduced islands of soft tissue, osteoid or lower new bone (BS/TS) created at this locations, as demonstrated by a previous report [6].

By inhibiting MMPs activities, doxycycline could indirectly prevent activation of cytokines such as TGF-β that could be associated with BMP-2 signaling of bone formation [57]. The reduction of BMP-2 was proven in the presence of doxycycline [55,58]. Moreover, low bone density complies with poor bone quality that refers to the weak bone tissue ensemble of structure and architectural properties [59], which may lead to further bone resorption [60]. Newly formed bone tissue may have a lower mineral content than the pre-existing older bone tissue; a great collagen production is warranted [61]. We speculate that the active trabecular bone remodelling might be affected, due to the low number of osteoclast recruitment (Table 5 and Table 6) (Figure S5) and the loss of doxycycline activity, which allow the progressive bone reduction over time, as reported [62]. An additional biochemical analysis, through Raman spectroscopy, would probably help to understand this point. The M1/M2 ratio is defined as an important factor determining membrane integration. Shifting this ratio or increasing M2 macrophages number is a potential strategy to promote bone repair [20]. Tough immune modulators, administered through biomaterials promoting M2 (IL-4)-like phenotypes, are currently being studied, it must be considered that M2-type macrophages are also involved in fibrotic diseases development [50]; thereby, this attempt should be adopted with caution. 

To the best of our knowledge, this is the first study to elucidate the potential of Zn-HOOC-Si-Membranes to enhance osteogenesis, angiogenesis, and osteoimmunomodulation for guide bone regeneration. This work is promising but preliminary, and some limitations need to be addressed before clinical application. A limitation of the present research is the relatively small sample size. This study should also be undertaken at different time points. A positive control group (like a group treated with a commercially available membrane) could also have been considered. 

In vitro studies on molecular mechanisms and biomarkers analyses are required, in order to verify bone regeneration engineering efficiency for clinical applications. Several limitations were encountered in the rabbit model that jeopardize the methodology, as the lack of fixation devices to stabilize the clot and potentially bone damage due to the animal high mobility [63]. Finally, the simultaneous application of zinc and silica in Zn-HOOC-Si-Membranes made it challenging to interpret the results, in terms of determining the factor most strongly associated with positive outcomes of the attained bone regeneration, or if there exists a combined action of both elements. Zn-HOOC-Si polymeric membranes, created new bone in close contact with the biomaterial, producing a significant increase in blood vessels and a bridge-like figures, joining up different areas of the new bone (Table S4). It increases interconnectivity and bone thickness, as both are considered as signs of bone regeneration [63]. This nanostructured bio-engineered material, which was performed as a net mechanical barrier that prevented soft tissue from growing into the bony defect, before osteogenic cells were able to seal and develop new bond matrices for bone regeneration; thereby, the presented membranes accomplished the required properties [64] for being a biomaterial for guided bone regeneration. The novelty of our work lies in the fact that it has been demonstrated that elongation of macrophages has promoted a pro-healing phenotype in rabbit bone defects, treated with Zn-HOOC-Si polymeric nanostructured membranes, which perform as a bioactive modulator for signals transferred to the surgical defect. These outcomes support future studies, which could lead to novel alternatives for reconstructions of the maxillofacial skeleton. Ongoing experiments using large animal models, including the application of experimental membranes for guided tissue regeneration should be proposed. 

## 5. Conclusions

All membranes promoted a generalized bone healing at the rabbit calvarial defect. Both Zn-HOOC-Si-Membranes and Dox-HOOC-Si-Membranes showed the lowest ratio M1/M2, modulating the macrophages polarization toward pro-healing phenotypes. Zn-HOOC-Si-Membranes enhanced biological activity, attained a balanced remodeling, and achieved the greatest regenerative efficiency after osteogenesis and angiogenesis assessments. The bone-integrated Zn-HOOC-Si-Membranes may contribute to novel therapeutic approaches and can be considered as bioactive modulators, which provoke a M2 macrophages increase, being a potential strategy for promoting bone repair.

## Figures and Tables

**Table 1:** Histomorphometric data obtained within the new bone created at the region of interest after Von Kossa staining (Mean ± Standard error).

	BS (mm^2^)	BS/TS (%)	OS/TS (%)	BPm (mm)	BTh (mm)
**HOOC-Si**	6.678 ± 0.708	51.65 ± 3.14	6.83 ± 0.34	122.421 ± 8.015	1.185 ± 0.068
**Zn-HOOC-Si**	7.106 ± 0.641	58.56 ± 3.32	6.72 ± 0.41	116.789 ± 7.660	1.249 ± 0.778
**Dox-HOOC-Si**	5.985 ± 0.587	54.03 ± 2.81	7.78 ± 0.49	109.758 ± 5.673	1.158 ± 0.567
**Control**	6.063 ± 0. 130	46.40 ± 4.42	7.07 ± 0.63	93.765 ± 7.018	1.053 ± 0.701

**Table 2:** Statistical results (*p* values) after pairwise comparisons. Bold numbers mean significance at *p* < 0.05.

	BS/TS	OS/TS	BPm	BTh
**HOOC-Si Zn-HOOC-Si**	**0.032**	0.822	0.589	0.571
**HOOC-Si Dox-HOOC-Si**	0.128	0.091	0.295	0.925
**HOOC-Si Control**	0.558	0.783	**0.020**	0.192
**Zn-HOOC-Si Dox-HOOC-Si**	0.428	**0.010**	0.301	0.256
**Zn-COOH-Si Control**	**0.034**	0.524	**0.004**	0.037
**Dox-COOH-Si Control**	**0.044**	0.216	**0.041**	0.201

Abbreviations: BS: Bone Surface; OS: Osteoid Surface; TS: Total Surface; BPm: Bone Perimeter; BTh: Bone Thickness; Si: silica; Zn: zinc; Dox: doxycycline.

**Table 3:** Histomorphometric data acquired by fluorescence with calcein, within the new bone created at the region of interest (Mean ± Standard Error).

	BA/TA (%)	OA/TA (%)	OPm (mm)	BPm (mm)
**COOH-Si**	84.25 ± 1.17	15.75 ± 1.17	351.99 ± 47.89	389.19 ± 49.99
**Zn-COOH-Si**	85.07 ± 1.91	19.38 ± 2.67	445.84 ± 80.21	567.27 ± 70.51
**Dox-COOH-Si**	86.36 ± 1.09	13.64 ± 1.10	284.24 ± 44.59	349.86 ± 38.48
**Control**	71.90 ± 3.98	13.07 ± 1.65	265.83 ± 45.26	258.83 ± 39.79

**Table 4:** Statistical results (*p* values) after pairwise comparisons. Bold numbers mean significance at *p* < 0.05.

	BA/TA	OA/TA	OPm	BPm
**COOH-Si Zn-COOH-Si**	0.693	0.228	0.366	**0.044**
**COOH-Si Dox-COOH-Si**	0.341	0.341	0.334	0.600
**COOH-Si Control**	**0.004**	0.351	0.330	0.126
**Zn-COOH-Si Dox-COOH-Si**	0.313	0.115	0.161	**0.025**
**Zn-COOH-Si Control**	**0.002**	**0.005**	0.134	**0.002**
**Dox-COOH-Si Control**	**0.003**	0.706	0.717	**0.035**

Abbreviations: TA: Total Area; OA: Osteoid Area; OPm: Osteoid Perimeter; BA: Bone Area; BPm: Bone Perimeter; Si: silica; Zn: zinc; Dox: doxycycline.

**Table 5:** Bone cells and blood vessels counts (units/mm^2^) within the new bone created at the region of interest (Mean ± Standard Error).

	Osteocytes/mm^2^	Osteoblasts/mm^2^	Osteoclasts/mm^2^	Blood Vessels/mm^2^
**HOOC-Si**	1020.913 ± 45.860	271.959 ± 19.221	113.736 ± 15.773	14.983 ± 0.895
**Zn-HOOC-Si**	1030.916 ± 42.915	305.997 ± 16.518	107.693 ± 12.058	17.769 ± 1.059
**Dox-HOOC-Si**	969.455 ± 38.792	316.161 ± 20.403	98.818 ± 12.058	14.714 ± 0.686
**Control**	875.207 ± 37.083	244.811 ± 15.767	152.445 ± 24.204	14.071 ± 0.941

**Table 6:** Statistical results (*p* values) after pairwise comparisons. Bold numbers mean significance at *p* < 0.05.

	Osteocytes/mm^2^	Osteoblasts/mm^2^	Osteoclasts/mm^2^	Blood Vessels/mm^2^
**HOOC-Si Zn-HOOC-Si**	0.845	0.162	0.771	**0.036**
**HOOC-Si Dox-HOOC-Si**	0.609	0.131	0.105	0.824
**HOOC-Si Control**	**0.005**	0.397	0.662	0.472
**Zn-HOOC-Si Dox-HOOC-Si**	0.365	0.398	0.097	**0.012**
**Zn-HOOC-Si Control**	**0.001**	**0.007**	0.612	**0.003**
**Dox-HOOC-Si Control**	**0.036**	**0.014**	**0.041**	0.548

Abbreviations: Si: Silica; Zn: zinc; Dox: doxycycline.

**Table 7:** Macrophages counts (cells/mm^2^) and characterization by histomorphometric profile at the region of interest (Mean ± Standard Error).

	M1/mm^2^	M2/mm^2^	M1/M2
**HOOC-Si**	486.659 ± 64.096	474.123 ± 69.345	4.19 ± 0.74
**Zn-HOOC-Si**	304.801 ± 45.344	572.443 ± 56.740	1.03 ± 0.18
**Dox-HOOC-Si**	385.888 ± 52.769	617.890 ± 63.820	1.12 ± 0.22
**Control**	534.558 ± 57.845	286.359 ± 35.122	5.67 ± 1.21

**Table 8:** Statistical results (*p* values) after pairwise comparisons. Bold numbers mean significance at *p* < 0.05.

	M1/mm^2^	M2/mm^2^	M1/M2
**HOOC-Si Zn-HOOC-Si**	**0.037**	0.231	**0.000**
**HOOC-Si Dox-HOOC-Si**	0.213	**0.04**	**0.002**
**HOOC-Si Control**	0.590	**0.014**	0.350
**Zn-HOOC-Si Dox-HOOC-Si**	0.380	0.379	0.753
**Zn-HOOC-Si Control**	**0.004**	**0.000**	**0.000**
**Dox-HOOC-Si Control**	**0.040**	**0.000**	**0.002**

Abbreviations: Si: Silica; Zn: zinc; Dox: doxycycline; M1: M1 macrophages; M2: M2 polarized macrophages.

## Supplementary Materials

The following are available online at https://www.mdpi.com/2073-4360/12/5/1201/s1, Figure S1: For each lesion, a volume of interest (VOI) was selected to capture the bone defect localization, Figure S2: Sub-volumes were arranged in 9 regions in the same way as the Pmod Mini-VOIs, MiniVOI *BoneJ* Macro, within the bone defect when the HOOC-Si-Membrane (a), Zn-HOOC-Si-Membrane (b) and Dox-HOOC-Si-Membrane (c) were placed covering the defect. Reslices, crops and analyses HR reconstructions for each specific VOI (1–9) were obtained. Average branch length (ABRLE), Maximum branch length (MXBRLE), Total volume (TV) and Trabecular thickness maximum (TBTHMX) were measured. The images show differences between the inner and outer areas within the region of interest. Arrow heads point out the Mini-voids. Asterisks (*) are trabecular bone. Island-like bone formations correspond with pointers. Single arrows point at new bone areas, Figure S3: Representative peripheral micro-computed tomography (Micro-CT) in 2-D dimensions 45u resolution of the bone defect in the HOOC-Si-Membranes group. The scan plane 193th is shown. Arrow heads point out the edge defects. Single pointers correspond with soft tissue. Double pointers are membranes. Asterisks (*) are old trabecular bone. Island-like bone formations correspond with faced pointers. Single arrows are new bone. Interconnected ossified trabeculae and branches are signaled by double arrows. Bony bridging formations are identified with faced arrows, Figure S4: Representative peripheral micro-computed tomography (Micro-CT) in 2-D dimensions 30u resolution of the bone defect in the Zn-HOOC-Si-Membranes group. The scan plane 117th (a), 299th (b), 310th (c) and 325th (d) are presented. High branch length and interconnectivity may be seen. Arrow heads point out the edge defects. Single pointers correspond with soft tissue. Double pointers are the membrane. Asterisks (*) are old trabecular bone. Island-like bone formations correspond with faced pointers. Single arrows are new bone. Interconnected ossified trabeculae and branches are signaled by double arrows. Bony bridging formations are identified with faced arrows, Figure S5: Representative peripheral micro-computed tomography (Micro-CT) in 2-D dimensions 30u resolution of the bone defect in the Dox-HOOC-Si-Membranes group. The scan plane 0th (a), 53th (b), 82th (c), 189th (d), 272 th (e), and 313 th (f) are displayed. The images show differences between the inner and outer areas within the region of interest, through subsequent scan planes. There is scarce new formed bone within the defect which is limited by the surrounding healthy bone, or old bone (S5a). Bone formation occurred mainly from the periphery (S5b,c,f) of the defect, leaving the center of the lesion almost empty (S5d,e). Arrow heads point out the edge defects. Double arrows correspond with soft tissue. Asterisks (*) are old trabecular bone. Island-like bone formations correspond with pointers. Single arrows point at new bone areas, Figure S6: Representative peripheral micro-computed tomography (Micro-CT) in 2-D dimensions 30u resolution of the bone defect in the control group. The scan plane 183th is shown. The bone tissues at the defect showed scarce regeneration within the region of interest, limited to the margins, where minor new bone formation was shown. Arrow heads point out the edge defects. Double arrows correspond with soft tissue. Asterisks (*) are old trabecular bone. Island-like bone formations correspond with pointers. Single arrows point out new bone areas, Table S1: Data from the Micro-CT: *p* values from comparisons were obtained by using *BoneJ*, a free plugin for *ImageJ*. An *ImageJ* script was created to automatically perform the analysis on all subvolumes using the same HU density threshold, and BoneJ settings using crop (central lesion region) and threshold (changes in bone density). Analysis v5 [high crop (300px) and low threshold (500)], at 75 px, was selected for the analysis. Bold numbers indicate significance at *p* < 0.05, Table S2: Hunsfield density MicroCT data from total miniVOIs analysis (Mean ± Standard Error), Table S3: Statistical results (*p* values) after pairwise comparisons. Bold numbers indicate significance at *p* < 0.05, Table S4: Hunsfield density MicroCT data from central miniVOIs analysis (Mean ± Standard Error), Table S5: Statistical results (*p* values) after pairwise comparisons. Bold numbers indicate significance at *p* < 0.05, Table S6: Hunsfield density MicroCT data from periphery miniVOIs analysis (Mean ± Standard Error), Table S7: Statistical results (*p* values) after pairwise comparisons. Bold numbers indicate significance at *p* < 0.05.
