# Supplementary material for: Doxycycline and Zinc Loaded Silica-Nanofibrous Polymers as Biomaterials for Bone Regeneration
Source: Polymers (Basel). 2020 May 25;12(5):1201. doi: 10.3390/polym12051201 (PMC7285172; doi:10.3390/polym12051201)
Supplement: Supplementary file 1 [file polymers-12-01201-s001.pdf]

polymers

Article

Doxycycline and and zinc loaded silica-nanofibrous polymers as biomaterials for bone regeneration

Manuel Toledano[1], Manuel Toledano-Osorio[1], Raquel Osorio[1,*], Álvaro Carrasco-Carmona [1], José L. Gutiérrez-Pérez [2], Aida Gutiérrez-Corrales [2], María A. Serrera-Figallo [2], Christopher D. Lynch [3] and Daniel Torres-Lagares [2]

[1] University of Granada, Faculty of Dentistry, Dental Materials Section. Colegio Máximo de Cartuja s/n, Granada 18071, Spain; toledano@ugr.es (M.T.); mtoledano@correo.ugr.es (M.T.-O.); alcarcar94@correo.ugr.es (Á.C.-C.)

[2] University of Sevilla, Faculty of Dentistry, Oral Surgery Section. Avicena s/n, Sevilla 41009, Spain; jlgp@us.es (J.L.G.-P.); agcorrales@us.es (A.G.-C.); maserrera@us.es (M.A.S.-F.); danieltl@us.es (D.T.-L.)

[3] University Dental School & Hospital/University College Cork, Wilton, Cork, Ireland; chris.lynch@ucc.ie (C.D.L.)

* Correspondence: rosorio@ugr.es (R.O.) Tel.: +34-958243789

polymers

MDPI

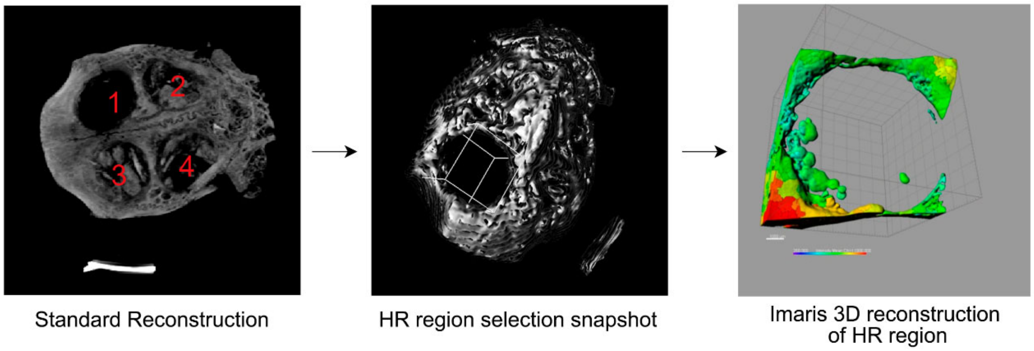

Figure S1. For each lesion, a volume of interest (VOI) was selected to capture the bone defect localization.

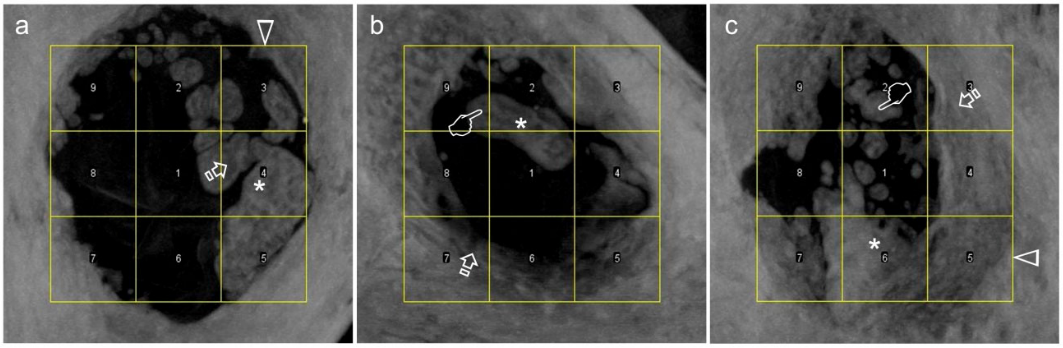

Figure S2. Sub-volumes were arranged in 9 regions in the same way as the Pmod Mini-VOIs, MiniVOI BoneJ Macro, within the bone defect when the HOOC-Si-Membrane (a), Zn-HOOC-Si-Membrane (b) and Dox-HOOC-Si-Membrane (c) were placed covering the defect. Reslices, crops and analyses HR reconstructions for each specific VOI (1-9) were obtained. Average branch length (ABRLE), Maximum branch length (MXBRLE), Total volume (TV) and Trabecular thickness maximum (TBTHMX) were measured. The images show differences between the inner and outer areas within the region of interest. Arrow heads point out the Mini-voids. Asterisks (*) are trabecular bone. Island-like bone formations correspond with pointers. Single arrows point at new bone areas.

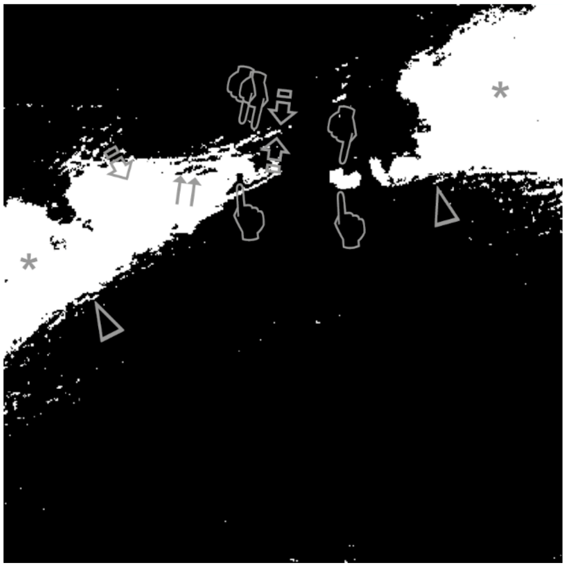

Figure S3. Representative peripheral micro-computed tomography (Micro-CT) in 2-D dimensions 45u resolution of the bone defect in the HOOC-Si-Membranes group. The scan plane 193th is shown. Arrow heads point out the edge defects. Single pointers correspond with soft tissue. Double pointers are membranes. Asterisks (*) are old trabecular bone. Island-like bone formations correspond with faced pointers. Single arrows are new bone. Interconnected ossified trabeculae and branches are signaled by double arrows. Bony bridging formations are identified with faced arrows.

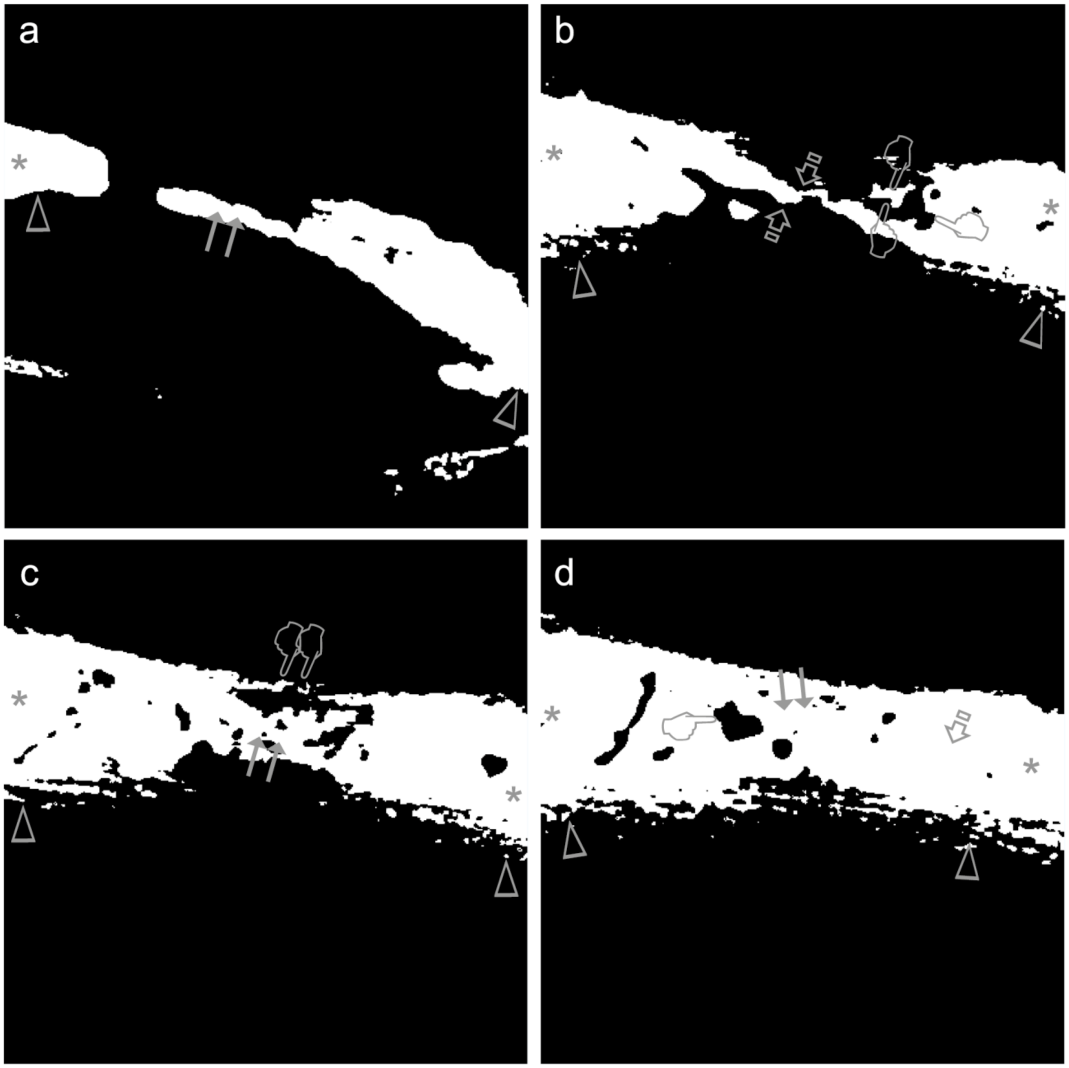

Figure S4. Representative peripheral micro-computed tomography (Micro-CT) in 2-D dimensions 30u resolution of the bone defect in the Zn-HOOC-Si-Membranes group. The scan plane 117th (a), 299th (b), 310th (c) and 325th (d) are presented. High branch length and interconnectivity may be seen. Arrow heads point out the edge defects. Single pointers correspond with soft tissue. Double pointers are the membrane. Asterisks (*) are old trabecular bone. Island-like bone formations correspond with faced pointers. Single arrows are new bone. Interconnected ossified trabeculae and branches are signaled by double arrows. Bony bridging formations are identified with faced arrows.

polymers
MDPI

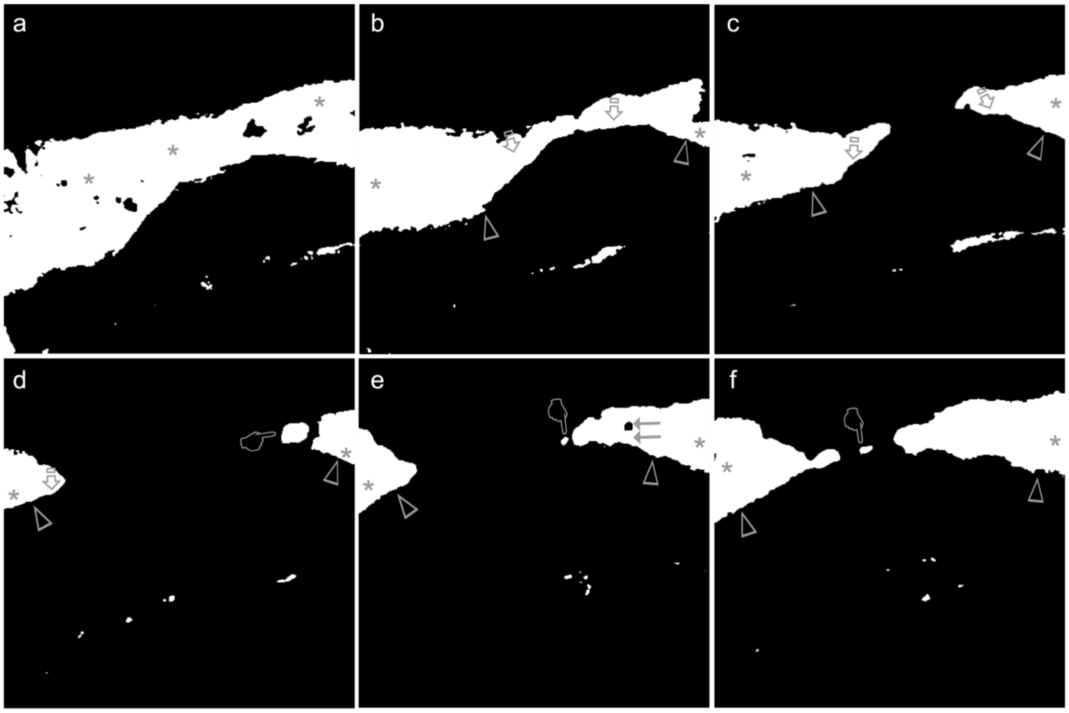

Figure S5. Representative peripheral micro-computed tomography (Micro-CT) in 2-D dimensions 30u resolution of the bone defect in the Dox-HOOC-Si-Membranes group. The scan plane 0th (a), 53th (b), 82th (c), 189th (d), 272 th (e), and 313 th (f) are displayed. The images show differences between the inner and outer areas within the region of interest, through subsequent scan planes. There is scarce new formed bone within the defect which is limited by the surrounding healthy bone, or old bone (S5a). Bone formation occurred mainly from the periphery (S5b, S5c, S5f) of the defect, leaving the center of the lesion almost empty (S5d, S5e). Arrow heads point out the edge defects. Double arrows correspond with soft tissue. Asterisks (*) are old trabecular bone. Island-like bone formations correspond with pointers. Single arrows point at new bone areas.

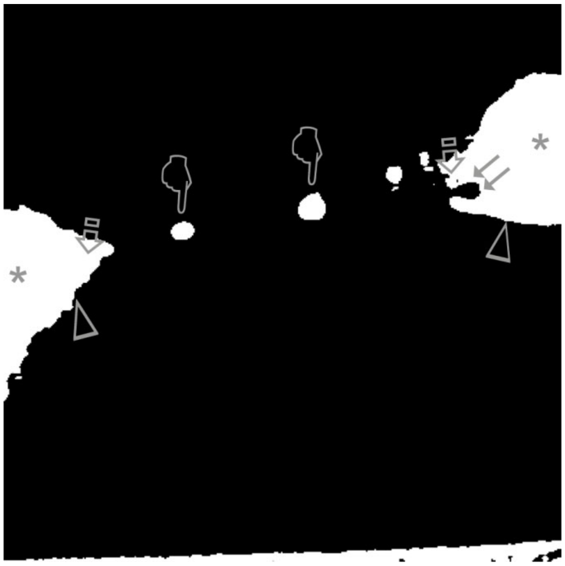

Figure S6. Representative peripheral micro-computed tomography (Micro-CT) in 2-D dimensions 30u resolution of the bone defect in the control group. The scan plane 183th is shown. The bone tissues at the defect showed scarce regeneration within the region of interest, limited to the margins, where minor new bone formation was shown. Arrow heads point out the edge defects. Double arrows correspond with soft tissue. Asterisks (*) are old trabecular bone. Island-like bone formations correspond with pointers. Single arrows point out new bone areas.

Table S1. Data from the Micro-CT: P values from comparisons were obtained by using BoneJ, a free plugin for ImageJ. An ImageJ script was created to automatically perform the analysis on all subvolumes using the same HU density threshold, and BoneJ settings using crop (central lesion region) and threshold (changes in bone density). Analysis v5 [high crop (300px) and low threshold (500)], at 75 px, was selected for the analysis. Bold numbers indicate significance at $P < 0.05$.

	ABRLE	MXBRLE	TV	TBTHMX
**HOOC-Si Dox-HOOC-Si**	0.116	**0.037**	**0.083**	**0.071**
**Zn-HOOC-Si Dox-HOOC-Si**	0.185	**0.011**	**0.083**	0.143
**Dox-COOH-Si Control**	**0.077**	0.327	**0.002**	0.234

Abbreviations: Si: Silica; Zn: zinc; Dox: doxycycline; ABRLE: Average branch length; MXBRLE: Maximum branch length; TV: Total volume; TBTHMX: Trabecular thickness maximum.

.

Table S2a. Hunsfield density MicroCT data from total miniVOIs analysis (Mean±Standard Error).

	Normalized Density	Total Average Vol x 1000	Hot Average
HOOC-Si	0.25± 0.03	5.99 ± 0.66	744.73 ± 48.70
Zn-HOOC-Si	0.26± 0.02	6.17 ± 0.52	805.65 ± 49.15
Dox-HOOC-Si	0.19± 0.03	4.66 ± 0.63	686.19 ± 71.01
Control	0.27± 0.03	6.42 ± 0.80	744.03 ± 67.60

Table S2b. Statistical results (*P* values) after pairwise comparisons. Bold numbers indicate significance at $P < 0.05$.

	Normalized Density	Total Average Vol x 1000	Hot Average
**HOOC-Si Zn-HOOC-Si**	0.768	0.797	0.296
**HOOC-Si Dox-HOOC-Si**	**0.098**	**0.092**	0.468
**HOOC-Si Control**	0.635	0.643	0.993
**Zn-HOOC-Si Dox-HOOC-Si**	**0.048**	**0.047**	**0.078**
**Zn-COOH-Si Control**	0.805	0.793	0.450
**Dox-COOH-Si Control**	**0.049**	**0.047**	0.461

Abbreviations: Vol: volume; Si: silica; Zn: zinc; Dox: doxycycline.

Table S3a. Hunsfield density MicroCT data from central miniVOIs analysis (Mean±Standard Error).

	Normalized Density	Total Average Vol x 1000	Hot Average
**HOOC-Si**	0.16 ± 0.05	3.97 ± 1.32	594.10 ± 158.80
**Zn-HOOC-Si**	0.06 ± 0.01	1.52 ± 0.23	312.82 ± 81.48
**Dox-HOOC-Si**	0.06 ± 0.03	1.40 ± 0.67	-64.66 ± 203.74
**Control**	0.16 ± 0.08	3.78 ± 1.95	522.18 ± 133.41

Table S3b. Statistical results (*P* values) after pairwise comparisons. Bold numbers indicate significance at $P < 0.05$.

		Normalized Density	Total Average Vol x 1000	Hot Average
**HOOC-Si**	**Zn-HOOC-Si**	0.112	0.121	0.138
**HOOC-Si**	**Dox-HOOC-Si**	0.136	0.136	0.072
**HOOC-Si**	**Control**	0.942	0.926	0.763
**Zn-HOOC-Si**	**Dox-HOOC-Si**	0.884	0.866	0.157
**Zn-COOH-Si**	**Control**	0.302	0.300	0.250
**Dox-COOH-Si**	**Control**	0.170	0.165	**0.006**

Abbreviations: Vol: volume; Si: silica; Zn: zinc; Dox: doxycycline.

Table S4a. Hunsfield density MicroCT data from periphery miniVOIs analysis (Mean±Standard Error).

	Normalized Density	Total Average Vol x 1000	Hot Average
**HOOC-Si**	0.24 ± 0.03	5.84 ± 0.73	759.91 ± 55.76
**Zn-HOOC-Si**	0.26 ± 0.02	6.24 ± 0.59	797.61 ± 53.97
**Dox-HOOC-Si**	0.15 ± 0.02	3.69 ± 0.53	641.33 ± 80.04
**Control**	0.23 ± 0.03	5.76 ± 0.84	722.86 ±78.30

Table S4b. Statistical results (*P* values) after pairwise comparisons. Bold numbers indicate significance at $P < 0.05$.

		Normalized Density	Total Average Vol x 1000	Hot Average
**HOOC-Si**	**Zn-HOOC-Si**	0.568	0.557	0.600
**HOOC-Si**	**Dox-HOOC-Si**	**0.005**	0.188	**0.005**
**HOOC-Si**	**Control**	0.926	0.705	0.922
**Zn-HOOC-Si**	**Dox-HOOC-Si**	**0.001**	**0.042**	**0.001**
**Zn-COOH-Si**	**Control**	0.601	0.421	0.621
**Dox-COOH-Si**	**Control**	**0.029**	0.370	**0.028**

Abbreviations: Vol: volume; Si: silica; Zn: zinc; Dox: doxycycline.
